# Illumina WG-6 BeadChip strips should be normalized separately

**DOI:** 10.1186/1471-2105-10-372

**Published:** 2009-11-11

**Authors:** Wei Shi, Ashish Banerjee, Matthew E Ritchie, Steve Gerondakis, Gordon K Smyth

**Affiliations:** 1 Bioinformatics Division, The Walter and Eliza Hall Institute of Medical Research, 1G Royal Parade, Parkville, VIC 3052, Australia; 2 Burnet Institute, 85 Commercial Road, Melbourne, VIC 3004, Australia

## Abstract

**Background:**

Illumina Sentrix-6 Whole-Genome Expression BeadChips are relatively new microarray platforms which have been used in many microarray studies in the past few years. These Chips have a unique design in which each Chip contains six microarrays and each microarray consists of two separate physical strips, posing special challenges for precise between-array normalization of expression values.

**Results:**

None of the normalization strategies proposed so far for this microarray platform allow for the possibility of systematic variation between the two strips comprising each array. That this variation can be substantial is illustrated by a data example. We demonstrate that normalizing at the strip-level rather than at the array-level can effectively remove this between-strip variation, improve the precision of gene expression measurements and discover more differentially expressed genes. The gain is substantial, yielding a 20% increase in statistical information and doubling the number of genes detected at a 5% false discovery rate. Functional analysis reveals that the extra genes found tend to have interesting biological meanings, dramatically strengthening the biological conclusions from the experiment. Strip-level normalization still outperforms array-level normalization when non-expressed probes are filtered out.

**Conclusion:**

Plots are proposed which demonstrate how the need for strip-level normalization relates to inconsistent intensity range variation between the strips. Strip-level normalization is recommended for the preprocessing of Illumina Sentrix-6 BeadChips whenever the intensity range is seen to be inconsistent between the strips. R code is provided to implement the recommended plots and normalization algorithms.

## Background

**Figure 1:**
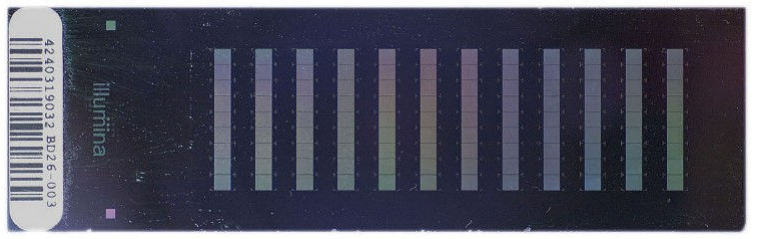
**Physical layout of twelve equally-spaced strips in a Illumina Sentrix-6 BeadChip**. Each array is made up of a pair of strips, one-below the other.

Illumina Whole-Genome Expression BeadChips have been widely adopted for high-throughput gene expression analysis in the past few years. Most popular and comprehensive of these are the HumanWG-6 and MouseWG-6 (Sentrix-6) BeadChips. Each Sentrix-6 BeadChip allows the interrogation of six RNA samples in parallel and produces data that can be treated as coming from six independent microarrays. Physically, each Sentrix-6 BeadChip consists of twelve equally-spaced strips of beads (Figure 1). Each pair of adjacent strips comprises a single microarray and is hybridized with a single RNA sample. In the first generation of Human-6 and Mouse-6 BeadChips, the first strip of each array was populated mainly with probes for curated RefSeq transcripts while the second strip was populated with probes for less well-annotated transcripts from a variety of databases including RefSeq, RIKEN FANTOM, Gnomon and Unigene [1].

Normalization of microarray data is an essential analysis step which removes unwanted technical variation and ensures that expression values from different arrays are comparable. Normalization needs to adjust not only for technical variation between RNA samples but also for systematic measurement errors associated with the hybridizing and scanning steps. In the case of Illumina BeadChips, normalization needs to adjust for technical variation within each BeadChip, including spatial variation across the slide.

All normalization strategies proposed so far for Illumina BeadChips carry out normalization at the array level, meaning that data from the two strips comprising each array are combined before normalization is conducted. The simplest and most popular strategy is to log-transform the probe-wise expression summaries for each array which are then quantile normalized between arrays [2-5]. This strategy normalizes the arrays, but fails to take any account of variation between the two strips comprising each array, despite the fact that the physical spacing between strips is the same as the spacing between arrays.

This study illustrates by way of a data example that between-strip technical variation can be substantial. We demonstrate that normalizing at the strip level rather than at the array level can effectively remove this between-strip variation, improve the precision of gene expression measurements and discover more differentially expressed (DE) genes. Many of the extra genes found are functionally closely related to the knockout genes in the experiment.

## Results and Discussion

### Strip-level variation

As an illustrative case study, we consider data from an experiment involving 18 RNA samples hybridized to Illumina Mouse-6 Version 1.1 BeadChips. The data were generated as part of a study designed to dissect molecular components of the NF-*κ*B1 pathway which mediates the immune response to pathogen invasion. The experiment studied the response to LPS stimulation in macrophages from wild-type, Nfkb1 knockout and Tpl2 knockout mice. For each of the three genotypes, RNA samples were taken prior to LPS stimulation and 1 hour and 3 hours after LPS stimulation, making a total of nine experimental conditions (three genotypes by three times). Two biological replicates were conducted of the entire experiment, making a total of 18 RNA samples hybridized to three BeadChips. Illumina BeadStudio software was used to output a summary probe profile file containing a raw intensity value for each probe on each array of the experiment. The arrays contain 46,657 probes, so the raw data is a 46, 657 × 18 matrix of intensities.

**Figure 2:**
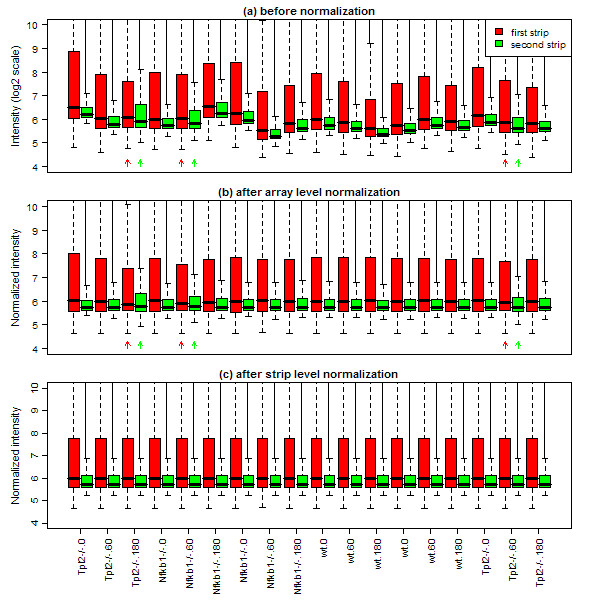
**Boxplots showing intensity distributions for each strip on each array**. Panel (a) shows raw data output from BeadStudio on the log_2 _scale. Panel (b) shows array-level quantile-normalized data. Panel (c) shows strip-level quantile-normalized data. The first six arrays from the left come from the first chip, the second six arrays from the second chip and last six arrays from the third chip. The first strip of each array is red and the second strip is green. The two strips belonging to each array have the same sample name. Log-intensities greater than 10 are omitted from the plot to better show the main body of values.

Figure 2(a) shows the distribution of probe intensities for each strip of each array. Each strip is shown as a separate box. The consecutive strips top-to-bottom down each BeadChip (as seen in Figure 1) are plotted left to right across the plot. Each array corresponds to two consecutive strips, so there are 36 boxplots in all. As expected, the first strip has higher average intensity than the second strip for every array. This is because probes on the first strip are derived from curated RefSeq transcripts which make up most of the moderate to highly expressed transcripts. By contrast, the second strip contains probes for many predicted or poorly annotated transcripts, which are less likely to be expressed in any individual sample. Indeed, the top quartile of the second strips is typically only about the same level as the median of the first strip on the same array. The first strips also show much wider intensity ranges, as evidenced by longer boxes in the plot, than the second strips.

Figure 2(a) shows noticeable variation in average intensity between arrays. This is not necessarily of any concern, as it will be removed by normalization. What are of concern are inconsistencies in the relativities between the two strips. In particular, there are several second-strips (indicated by green arrows) that show unusually large spread relative to the first-strips for the same arrays (indicated by red arrows). These three arrays show an unusually wide range of intensities on the second strip while the first strip is normal. This is a symptom of intra-array spatial variation on the BeadChips.

Figure 2(b) shows the distribution of normalized intensities after array-level quantile normalization. Array-level normalization ensures that the distribution of intensities is the same for each array but not for each strip. For some arrays, a wider range of intensities on the second strip before normalization forces a narrower range of intensities on the first strip after normalization, as the normalization process attempts to compensate. This is most noticeable for the three arrays noted above. For these arrays, the second strips still have wider than average spread after normalization, but now the corresponding first strips become the least spread of all the first strips. The intra-array variation in the raw intensities, interacting with the normalization method, has resulted in artificially compressed intensity values on the first strips for these arrays.

**Figure 3:**
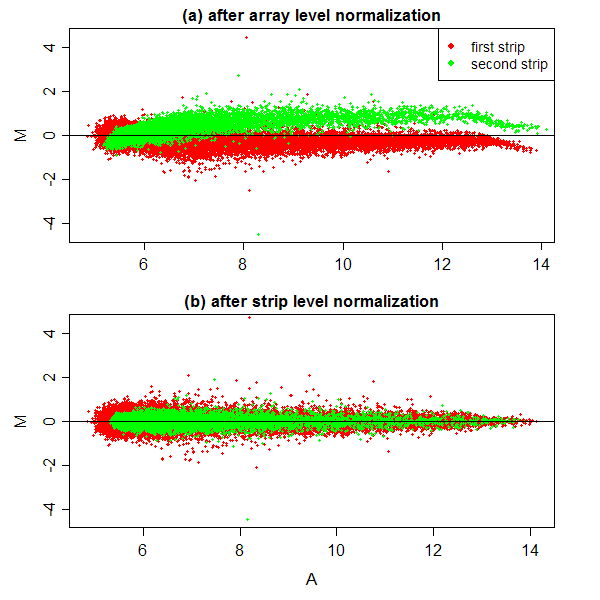
**MA-plots for two replicate arrays after (a) array-level normalization and (b) strip-level normalization**. Both arrays are hybridized with RNA from Tpl2^-/- ^macrophages after 180 mins. The horizontal axis shows A-values, the average log_2_-intensity for each probe, and the vertical axis shows M-values, the log-intensity ratio between the two replicate arrays for each probe. The horizontal zero line represents equality of probe intensities.

The same problem can be seen even more vividly by comparing replicate arrays. An MA-plot compares two arrays by plotting probe-wise log-ratios between the arrays against average log-intensities. The MA-plot in Figure 3(a) compares the third array in the experiment to the other array hybridized with RNA sample from the same source. Both arrays hybridize to the biological sample Tpl2^-/- ^180 mins. The plot shows that first-strip probes are systematically lower on the first replicate whereas the second-strip probes are systematically higher. This systematic bias has been introduced by the normalization. The bias is substantial, with the strip-1 and strip-2 bands in Figure 3(a) separated by more than one unit of the vertical axis, corresponding to a 2-fold discrepancy in intensity. This lack of alignment between the two strips is clearly systematic and is enormously greater than could arise from random variation. Array-level normalization produces here the fiction that the strip-2 probes appear generally up-regulated when in reality almost certainly most are not differentially expressed. Similar bias can be observed on other replicate pairs of arrays (data not shown).

A solution to the above problem is to normalize the first and second strips separately. In this approach, quantile normalization is applied twice, once to the 18 first strips and once to the 18 second strips. This forces all first strips to have the same overall intensity distribution, and similarly for the second strips, but no relationship between the first and second strips is assumed. Figure 2(c) displays the distribution of intensities after strip-level normalization. Strip-level normalization has the desirable property that the intensity distribution for each strip is equalized across arrays. In particular, the normalized intensities for one strip can no longer be influenced by aberrations on the other strip of the same array.

The success of strip-level normalization can be seen by comparing replicates, which now show high concordance. Figure 3(b) shows the third array and its replicate after strip-level normalization. The strip-1 and strip-2 bands are now perfectly aligned about the *M *= 0 line, i.e., the expression log-ratios are now randomly scattered about zero for both first and second strips. This shows that the bias related to the physical position of the strips within the arrays has been removed.

### Between-array precision

The most basic aim of normalization is to increase the consistency between replicate arrays. To examine this, we computed the standard deviation of normalized log_2_-expression values between replicate arrays for each probe. Each of the nine experimental conditions has a pair of replicate arrays, so each pooled standard deviation has nine degrees of freedom. When array-level normalization was used, the median of the probe-wise standard deviations was 0.155. This implies that replicate expression values have a typical variation up or down by about 11% on the unlogged scale (because 2^0.155 ^= 1.11). When strip-level normalization was used, the median standard deviation decreased more than 10% to 0.139. This corresponds to a 20% increase (0.155/0.139 squared) in statistical information, equivalent to the gain which would be achieved by increasing the number of microarrays used in the experiment by 20%. Since normalization can reduce only the technical component of the residual variability, not the biological component, we can conclude that the technical standard deviation corresponding to microarray precision has been reduced by substantially more than 10%.

### Differential expression analysis

Next we identified probes which are differentially expressed between wild-type and the two knockout lines. Significance analysis was conducted using empirical Bayes moderated *t*-statistics [6,7], a popular method which gains precision compared to an ordinary *t*-test by moderating the standard deviations across probes. Not surprisingly, the increased precision arising from strip-level normalization translates directly into greater numbers of DE probes. Table 1 gives the numbers of DE probes between the knockout sample and the wild-type sample at each time point. Twice as many DE probes can be detected at a 5% false discovery rate after strip-level normalization as compared to the usual array-level normalization. The larger list of DE probes after strip-level normalization includes almost all of those found after array-level normalization.

**Table 1:** Numbers of differentially expressed probes between knockout and wild-type samples at various times at a 5% false discovery rate

Normalization	Genotype					
	
	Tpl2^-/-^			Nfkb1^-/-^		
		
	0	60	180 mins	0	60	180 mins
Array-level	6(4)	20(19)	47(42)	23(23)	49(42)	140(135)
Strip-level	17	40	89	49	96	290

Numbers in parentheses are the number of probes which were also found by strip-level normalization.

**Figure 4:**
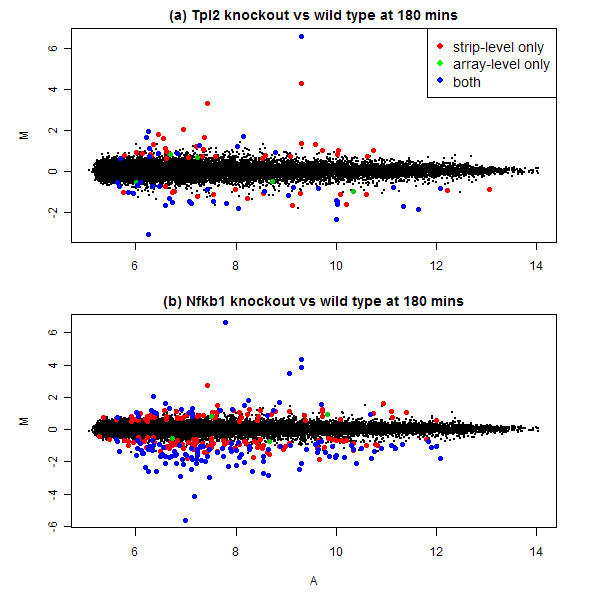
**MA-plots for DE probes discovered by different normalization strategies (a) Tpl2 knockout vs wild-type at 180 mins and (b) Nfkb1 knockout vs wild-type at 180 mins**. This figure highlights the log_2 _fold changes of DE probes discovered by different normalization strategies when comparing Tpl2^-/- ^with wild-type at 180 mins and comparing Nfkb1^-/- ^with wild-type at 180 mins. M-values (*log*_2_-fold-change between the two conditions) and A-values (average log_2_-intensity) were obtained from the linear models fitted to the probe intensities from strip-level normalization. Plots were generated using the plotMA function in the Bioconductor package limma. Probes in red, green and blue were discovered by strip-level normalization only, array-level normalization only, and both respectively. All other probes are in black. Numbers of DE probes can be found in Table 1.

Figure 4 shows fold-changes for the DE genes in the two knockout lines, log_2 _fold change on the vertical axis vs average log-intensity on the horizontal axis. DE genes are distributed more or less evenly across the whole intensity range. As expected, genes with the very largest fold changes were detected by both normalization methods, but the genes newly discovered by strip-level normalization were also amongst the larger fold-changes. For Tpl2^-/-^, the second, third and fourth ranked genes in terms of fold-change were found only by strip-level normalization. In fact, most of the genes up-regulated by the Tpl2 knockout are detected only after strip-level normalization. The few genes found only by array-level normalization tend to have small fold changes.

### Functional analysis

In the previous section, we have demonstrated that strip-level normalization gives a global and substantial improvement in precision for the microarray data. Naturally, the improved precision increases the number of statistically significant genes. Figures 2 and 3 demonstrate the mechanism by which this improved precision arises, by improving the alignment of the strips. We can be confident that improving precision by a proper analysis of the physical properties of the microarrays will in general improve the reliability of our biological results, although specific effects will of course depend on the data set. In this section we confirm that strip-level normalization does indeed return more biologically meaningful results for this particular data.

Our functional analysis consists of two parts. Firstly we used the functional annotation tool DAVID [8] to look for biologically interesting annotation categories amongst our DE genes. Secondly, we used the Ingenuity database [9] to show that the DE genes are enriched for genes known to interact with the genes knocked out in the experiment. In both cases, genes DE at the 180 min time point were used for the analysis.

Consider first the Tpl2 knockout. At a false discovery rate (FDR) cutoff of 0.05, no DAVID terms at all were found to be significantly enriched in the list of DE genes found by array-level normalization. After strip-level normalization, on the other hand, fifteen highly relevant terms were found to be significantly enriched (Table 2). All fifteen DAVID terms found by strip-level normalization were grouped by DAVID into one cluster of closely related categories. As can be seen from Table 2, this cluster relates to the Toll-like receptor signaling pathway, exactly the pathway which is being studied in this experiment, and all the terms are of obvious biological relevance. In summary, strip-level normalization returns easily interpretable and highly relevant biological results whereas array-level normalization returns no significant results at all.

**Table 2:** Functional analysis for DE genes at Tpl2^-/- ^180 mins from different normalization strategies

		Array-level		Strip-level	
			
Source	Term	Count	FDR	Count	FDR
INTERPRO	Four-helical cytokine, core	4	0.94	9	7.5E-7
SP_PIR_KEYWORDS	cytokine	5	0.21	9	1.6E-4
KEGG_PATHWAY	Cytokine-cytokine receptor interaction	5	0.84	10	0.001
KEGG_PATHWAY	Toll-like receptor signaling pathway	-	-	7	0.002
KEGG_PATHWAY	Jak-STAT signaling pathway	3	1.0	8	0.002
GOTERM_MF_ALL	cytokine activity	5	1.0	9	0.007
GOTERM_CC_ALL	extracellular region part	15	0.072	22	0.008
GOTERM_CC_ALL	extracellular space	14	1.0	21	0.008
GOTERM_CC_ALL	extracellular region	16	0.072	23	0.013
KEGG_PATHWAY	Regulation of autophagy	-	-	4	0.038
INTERPRO	Interferon alpha	-	-	4	0.039
GOTERM_MF_ALL	receptor binding	7	1.0	12	0.043
SMART	IFabd	-	-	4	0.043
SP_PIR_KEYWORDS	glycoprotein	17	0.16	24	0.048
SP_PIR_KEYWORDS	Secreted	10	0.2	14	0.048

The first column is the data source from which terms in the second column come. The third column gives the number of DE genes found by array-level normalization which are associated with the term in the same row in the second column. FDR (False Discovery Rate) from the enrichment test for these genes in all the DE genes found by array-level normalization is given in the fourth column (see METHODS for details). The fifth and sixth column are similar with the third and fourth column respectively except that DE genes examined are from strip-level normalization rather than from array-level normalization.

Now consider the Nfkb1 knockout (Table 3). In this case, array-level normalization did manage to find two Gene Ontology (GO) terms and two other keywords at a 5% FDR, however strip-level normalization found many more terms at much higher significance levels. The only term found by array-level normalization but not by strip-level normalization is "glycoprotein", which is an extremely common keyword, and has no obvious role specific to the particular molecular pathways being studied here. Most of the terms (13 out of 17) found by strip-level normalization were grouped by DAVID into the same functional cluster relating to cytokine activity and immune response. Again, strip-level normalization gave strong, clearly interpretable results, whereas array-level normalization gave very limited results at best.

**Table 3:** Functional analysis for DE genes at Nfkb1^-/- ^180 mins from different normalization strategies

		Array-level		Strip-level	
			
Source	Term	Count	FDR	Count	FDR
GOTERM_BP_ALL	immune system process	19	0.002	29	8.2E-5
GOTERM_BP_ALL	immune response	15	0.001	22	1.1E-4
SP_PIR_KEYWORDS	cytokine	9	0.01	13	3.3E-4
GOTERM_BP_ALL	cytokine metabolic process	4	0.96	9	0.005
GOTERM_BP_ALL	cytokine biosynthetic process	4	0.96	9	0.006
INTERPRO	Fos transforming protein	3	1.0	5	0.006
GOTERM_BP_ALL	cytokine production	5	0.88	10	0.01
GOTERM_BP_ALL	leukocyte activation	7	0.92	13	0.01
GOTERM_BP_ALL	hemopoietic or lymphoid organ development	7	0.89	14	0.01
GOTERM_BP_ALL	regulation of cytokine biosynthetic process	3	1.0	8	0.011
GOTERM_BP_ALL	immune system development	7	0.91	14	0.013
GOTERM_BP_ALL	cell activation	7	0.93	13	0.013
GOTERM_BP_ALL	hemopoiesis	7	0.94	13	0.014
GOTERM_BP_ALL	positive regulation of translation	3	1.0	7	0.014
GOTERM_BP_ALL	positive regulation of cellular biosynthetic process	3	1.0	7	0.019
KEGG_PATHWAY	Cytokine-cytokine receptor interaction	10	0.056	14	0.027
GOTERM_MF_ALL	cytokine activity	9	0.16	13	0.036
SP_PIR_KEYWORDS	glycoprotein	36	0.017	51	0.18

Further evidence of the biological relevance of genes found by strip-level normalization can be seen from a gene interaction analysis using Ingenuity Pathway Analysis (IPA) tool. Only 1.7% of genes on the microarrays are known to interact with Nfkb1. Yet 7% of genes found by strip-level normalization are known to interact with Nfkb1, and almost all of these are in fact directly interacting genes (Table 4). This enrichment for Nfkb1 interacting genes is highly significant (*P *= 4e-6). The enrichment is virtually the same if we restrict to genes found only by strip-level normalization and not by array-level normalization (*P *= 0.0068). On the other hand, no interacting genes were found by array-level normalization only.

**Table 4:** Numbers of Nfkb1 interacting genes discovered by the two normalization methods.

Normalization method	DE genes	Interacting	Directly interacting
Array-level only	2	0	0
Strip-level only	99	6	6
Both	111	9	7
Neither	19988	335	221
Total	20200	350	234

The rows give respectively genes found by array-level normalization only, strip-level normalization only, both or neither. The second column gives the numbers of genes differentially expressed between Nfkb1^-/- ^and wild-type at 180 mins found by different normalization methods. The third column gives the numbers of genes interacting with Nfkb1 in each set (row) of DE genes in the second columns. This interaction could be direct or indirect according to IPA. The fourth columns shows the corresponding numbers of directly interacting genes.

**Figure 5:**
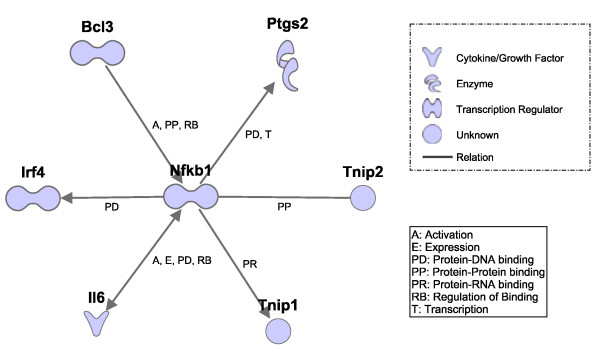
**Interactions between Nfkb1 and DE genes found only by strip-level normalization**. This gene interaction network is generated by Ingenuity Pathway Analysis software which uses a curated gene interaction database.

Six directly interacting genes were found by strip-level normalization only, and these are shown in an IPA display (Figure 5). Three are the transcriptional targets of Nfkb1: Interleukin 6 (Il6), Interferon regulatory factor 4 (Irf4) and Prostaglandin-endoperoxide synthase 2 (Ptgs2). Two of them have a protein-protein interaction with Nfkb1: B-cell leukemia/lymphoma 3 (Bcl3) and TNFAIP3 interacting protein 2 (Tnip2). TNFAIP3 interacting protein 1 (Tnip1) has a protein-RNA interaction with Nfkb1.

A literature search confirms that these six genes are direct targets of or associates with Nfkb1. Il6 is required for B- and T-cell growth and differentiation, neuronal and macrophage differentiation [10]. Inhibition of mouse Nfkb protein decreases expression of mouse Il6 mRNA in colonic tissue from mouse exhibiting experimentally induced colitis [11]. Irf4 is a lymphoid/myeloid-restricted member of the IRF transcription factor family that plays an essential role in the homeostasis and function of mature lymphocytes. Irf4 expression in HTLV-1-infected cells is driven through activation of the Nfkb and NF-AT pathways, resulting in the binding of p50, p65, and c-Rel to the Nfkb1 element and p50, c-Rel, and NF-ATp to the CD28RE element within the -617 to -209 region of the Irf4 promoter [12]. The proto-oncoprotein Bcl3 is a member of the Ikb family and is present predominantly in the nucleus. It acts as an adaptor between Nfkb p50/p52 and other transcription regulators [13].

This is far from a comprehensive study of the biological results of this experiment. Such a study will be published elsewhere, including RT-PCR confirmation of differential expression for selected genes and other follow-up assays. The bioinformatics analysis included here is however more than enough to demonstrate that the extra differential expression detected by strip-level normalization is biologically relevant and not merely a random sample of unrelated probes. Moreover the extra differential expression is sufficient to materially change the conclusions obtained.

### Probe filtering

As demonstrated above, inconsistencies in the relativities between the two strips caused array-level normalization to perform poorly. Now we want to investigate if it is possible to alleviate these inconsistencies by filtering out non-expressed probes. Probe filtering has been reported to be able to increase the power of detecting DE genes [14].

Probes with detection p-value greater than 0.1 on all arrays were deemed as non-expressed probes and filtered out. The intensity of a probe with detection p-value less than 0.1 will be greater than 90 percent of all negative controls [15]. In total, 22,505 non-expressed probes (48 percent of all probes) were identified. As expected, more such probes come from the second strip (60 percent of all non-expressed probes) than the first strip. However, inconsistencies in the relativities between the two strips clearly remain in the filtered raw data. Array-level normalization on the filtered raw data shows the same problem. Filtering probes after the normalization also fails to remove the inconsistencies [see Additional file 1: Supplemental Figures S1-S3].

By filtering out non-expressed probes, the number of DE probes increased for both normalization strategies. However, strip-level normalization still discovered about twice as many DE probes as compared to array-level normalization irrespective of whether probe filtering was performed before or after the normalization step (Tables 5 and 6). Functional analysis revealed that strip-level normalization still yields much more significant FDR scores for discovered DAVID terms than array-level normalization (Table 7 and Additional file 1: Supplemental Table S1). Two new DAVID terms ("response to other organism" and "antiviral defense") found by strip-level normalization, not array-level normalization, are closely related to this experiment. Put together, it was clearly demonstrated that probe filtering failed to remove intra-array variation.

**Table 5:** Number of differentially expressed probes between Tpl2^-/- ^knockout and wild-type samples at 180 mins at a 5% false discovery rate when probe filtering is applied or not applied

Normalization	Without filtering	With filtering	
		
		Before normalization	After normalization
Array-level	47	62	60
Strip-level	89	105	106

**Table 6:** Number of differentially expressed probes between Nfkb1^-/- ^knockout and wild-type samples at 180 mins at a 5% false discovery rate when probe filtering is applied or not applied

Normalization	Without filtering	With filtering	
		
		Before normalization	After normalization
Array-level	140	159	159
Strip-level	290	312	312

**Table 7:** Functional analysis for DE genes at Tpl2^-/- ^180 mins obtained from different normalization strategies when probe filtering is applied

		Array-level		Strip-level	
			
Source	Term	Count	FDR	Count	FDR
INTERPRO	Four-helical cytokine, core	7	1.7E-4	9	2.0E-6
SP_PIR_KEYWORDS	cytokine	8	2.9E-4	10	3.7E-5
KEGG_PATHWAY	Cytokine-cytokine receptor interaction	8	0.019	11	2.8E-4
KEGG_PATHWAY	Toll-like receptor signaling pathway	6	0.01	9	5.3E-5
KEGG_PATHWAY	Jak-STAT signaling pathway	6	0.054	9	5.7E-4
GOTERM_MF_ALL	cytokine activity	8	9.9E-3	10	1.1E-3
GOTERM_CC_ALL	extracellular region part	19	3.2E-3	24	5.6E-3
GOTERM_CC_ALL	extracellular space	18	4.1E-3	23	4.8E-3
GOTERM_CC_ALL	extracellular region	20	3.7E-3	25	0.01
KEGG_PATHWAY	Regulation of autophagy	3	0.4	5	2.8E-3
INTERPRO	Interferon alpha	3	0.81	4	0.037
GOTERM_MF_ALL	receptor binding	10	0.1	13	0.016
SMART	IFabd	3	0.57	5	1.2E-3
SP_PIR_KEYWORDS	glycoprotein	20	0.021	24	0.23
SP_PIR_KEYWORDS	Secreted	13	0.017	14	0.21
GOTERM_BP_ALL	response to other organism	6	0.24	8	0.013
SP_PIR_KEYWORDS	antiviral defense	3	0.38	5	4.1E-3

## Conclusion

It has been shown that strip-to-strip technical variation can have a material effect on expression values from Illumina BeadChips. If the usual strategy of array-level normalization is used, technical variation on one strip can artificially perturb the normalized intensity values on the other strip for the array, introducing bias, disagreement between replicates, and less precise assessment of differential expression. On the other hand, strip-level normalization successfully removes technical artifacts within as well as between arrays, solving all of the above problems and resulting in more powerful statistical analysis of the expression values.

The evidence we present consists of three major parts. Firstly we demonstrate that strip-level normalization gives a substantial improvement in precision for the microarray data. This improvement in precision is not restricted to individual genes, but is a global result affecting all the probes on the arrays. Secondly, we demonstrate by way of some carefully chosen plots, how this improvement arises by improving the alignment of the intensity values from the individual strips. The problem can be seen to arise from strips which have an unusually large or small intensity range (a long or short box in the boxplot) relative to their partner strip within the same array. We show that this phenomenon leads to mis-aligned bands for individual strips in MA-plots for replicate arrays. In this way, we have demonstrated the mechanism by which strip-level normalization leads to improved precision and removal of bias. Thirdly, we demonstrate by functional analysis that the extra genes discovered after strip-level normalization are not random but do indeed tend to be biologically related to the experimental conditions. Although this third component of our evidence is convincing, we view the first two components as the most compelling, because they demonstrate global results which affect all the probes on the arrays, not just those genes which happened to be significant in this experiment.

Detailed R code which should enable readers to repeat our analysis is provided in the Additional file 2. We also provide the strip-level normalization and plotting algorithms in the *illumina *package for R available from http://bioinf.wehi.edu.au/illumina/.

Our Figure 2 can be viewed as a "figure of merit" from which the need for strip-level normalization can be judged. In the Additional file 3, we provide R code for producing plots such as Figure 2. Our code is compatible with the Bioconductor project software including the lumi package for reading and pre-processing Illumina data. We recommend strip-level normalization whenever inconsistent boxplot inter-quartile ranges can be seen in this plot.

In our examples, we used what is probably the simplest and most popular normalization strategy for Illumina data. Our results are essentially the same using alternative preprocessing methods including model-based background correction [5,16], variance-stabilizing data transformation [17] or robust spline normalization [18]. The need for strip-level normalization remains.

Our data example used Version 1.1 MouseWG-6 BeadChips. Although Illumina stopped shipping this version of the BeadChip in 2008, data from this platform continues to be highly current. At our institution, Version 1.1 MouseWG-6 BeadChips continued to be hybridized until late in 2008. It is likely that the majority of experiments worldwide, which used human or mouse Version 1 WG-6 BeadChips, have not yet been published and so are still subject to primary analysis. The majority of Illumina BeadChip data which is publicly available through databases such as GEO [19] is Version 1.

The currently shipped versions of Illumina BeadChips (HumanWG-6 Version 3 or MouseWG-6 Version 2) no longer imbed separate sets of probes in the first and second strips of each array. Rather, the entire library of probes (bead types) is now randomly distributed over each strip. This means that the marked difference in intensity distribution between first and second strips on the Version 1 BeadChips is no longer apparent. However, the physical layout of strips on the chip is unchanged. Therefore the possibility of intra-array variation and the consequent need for strip-level normalization remains.

The probe-level expression summaries output by the Illumina BeadStudio software for HumanWG-6 Version 3 or MouseWG-6 Version 2 BeadChips do not provide any means to separate first and second strips. Expression values are already averaged over the two strips for each array. Instead, strip-level information can be obtained from the Bioconductor software package beadarray, which provides access to bead-level data summaries for Illumina BeadChips [20]. This process requires that the Illumina BeadScan software be configured to output bead-level data. With the new generation of BeadChips, strip-level normalization implies carrying out pre-processing and normalization of the two strips as if these were separate arrays. The potential need to analyse the strips separately has been noted by Dunning et al [4] although they do not elaborate on how this might occur. Our recommendation is that the two strips be treated as technical replicates in a differential expression analysis. The strip-level technical replication fits into the same format as developed previously for within-array replicate spots [21]. Software for utilizing these technical replicates in an differential expression analysis is readily available in the Bioconductor package limma, using the duplicateCorrelation function to estimate the correlation between the technical replicates [7]. While our experience with Version 2 and 3 BeadChips is limited so far, the potential for an improvement in precision is similar to that for Version 1 arrays when the spread of the intensity distribution is seen to vary considerably between strips, and bead-level analysis has demonstrated that spatial variation does occur on Version 2 and 3 BeadChips [22].

It is worth noting that the standard normalization method applied to data from Illumina's Infinium BeadChips for genotyping also occurs at the sub-array level. Similar to the WG-6 expression arrays, each Infinium array is made up of a number of strips (currently 2, 3 or 6 depending on the array type). Beads manufactured together in the same pool are located on a particular strip on each array and are normalized separately using a between channel/allele affine transformation [23]. The need for normalization at the strip-level rather than at the array-level is therefore essential for data from both genotyping and expression BeadChips.

## Methods

### Sample preparation and hybridization

Mice were obtained from three inbred lines, C57BL/6 (wild-type), an NF-*κ*B1 knockout line (Nfkb1^-/-^) [24] and a Tpl2 knockout line (Tpl2^-/-^) [25]. Bone marrow-derived macrophages (BMDMs) were generated as previously described [26]. BMDMs were stimulated with lipopolysaccharide (LPS) in culture to simulate response to a pathogen. Total cellular RNA was purified at different time points over a 3 hr period (0, 60 and 180 minutes) using the RNeasy Plus kit from Qiagen. Hybridization to Illumina Mouse-6 Version 1.1 BeadChips was conducted at the Australian Genome Research Facility using standard Illumina protocols. There were two biological replicates of the entire experiment, making a total of eighteen arrays on three BeadChips.

### Data processing

All analysis was undertaken using the R programming environment http://www.r-project.org. Probe summary profiles output by Illumina BeadStudio Version 3.0.14 software were read into R using the lumiR function of the lumi Bioconductor software package [18].

The position of each probe on first or second strip was determined by probe ID number. For MouseWG-6 Version 1 BeadChips, all probes on the second strip have probe ID numbers greater than 10^8^.

Raw summary expression data was log_2 _transformed and quantile normalized [27]. In array-level normalization, quantile normalization is applied to all the probes simultaneously. In strip-level normalization, quantile normalization is applied separately to (i) probes on the first strip of each array and (ii) to probes on the second strip. In other words, the first strips are normalized together as if the second strips did not exist, and then vice versa for the second strips.

Probe annotation, including gene symbols and Entrez Gene IDs, were obtained from the most recent Illumina bead manifest file for the MouseWG-6 BeadChips (Version 1.1, revision 3).

Differential expression analysis was conducted by fitting a linear model to the 18 microarrays and comparing the knockout genotypes with wild-type using empirical Bayes moderated *t*-statistics from the Bioconductor software package limma [6,7]. The probe-wise pooled standard deviations discussed in the results section were obtained as a by-product of the linear model, as the unmoderated residual standard deviations from the linear model.

### DAVID analysis

The National Institute of Health's DAVID tool (Database for Annotation, Visualization, and Integrated Discovery) uses a variant of Fisher's Exact test to test for enrichment of Gene Ontology terms or KEGG pathways in a list of genes [8]. DAVID reports false discovery rates (FDR) for each term or pathway, obtained by adjusting the p-values from Fisher's exact test by a method similar to that of Benjamini-Hochberg [28]. In this analysis, the enrichment test for each particular term in a list of DE genes is based on the number of DE genes associated with this term, the total number of DE genes, the number of background genes associated with this term and the total number of background genes. In our analysis, Entrez gene IDs were used as gene identifiers. A total of 19,482 Entrez gene IDs on the MouseWG-6 BeadChips were recognized by DAVID, and these were set as the background genes for the analysis. A FDR cutoff of 0.05 was used to identify significantly enriched categories.

### Ingenuity analysis

Ingenuity Pathway Analysis software [9] was used to find genes interacting with Nfkb1 or Tpl2. No interacting genes were found for Tpl2 from the list of DE genes either from array-level normalization or strip-level normalization. Genes found to be interacting with Nfkb1 can be found in Table 4, which were broken into four groups. Totally, 20,200 Entrez gene IDs from the chip were recognized by Ingenuity. 350 of them were found to be interacting with Nfkb1, which include both directly interacting genes and indirectly interacting genes. Fisher's Exact test was used to test the enrichment of Nfkb1 interacting genes.

## Authors' contributions

GKS and WS conceived the study. WS and MR performed the analysis and WS drafted the manuscript. SG and AB designed the biological experiment. AB performed the sample preparation. GKS finalized the manuscript. All authors read and approved the final manuscript.

## Supplementary Material

Additional file 1 **Supplemental document**. This file contains descriptions to R functions and analysis scripts used in this study and also the supplemental figures and tables for probe filtering. Click here for file

Additional file 2 **Analysis scripts**. This file contains R scripts used for performing the analysis in this study. Instructions on how to run these scripts can be found in Additional file 1. Click here for file

Additional file 3 **R functions**. This file includes the code of R functions which implement strip-level data retrieval, strip-level plotting and strip-level normalization. Instructions on how to use these functions can be found in Additional file 1. Click here for file
