# Supplementary material for: Illumina WG-6 BeadChip strips should be normalized separately
Source: BMC Bioinformatics. 2009 Nov 11;10:372. doi: 10.1186/1471-2105-10-372 (PMC2780421; doi:10.1186/1471-2105-10-372)
Supplement: Additional file 1 — Supplemental document. This file contains descriptions to R functions and analysis scripts used in this study and also the supplemental figures and tables for probe filtering. [file 1471-2105-10-372-S1.pdf]

Supplemental document

1 R functions

We have developed R functions for strip-level data retrieval, strip-level plotting and strip-level normalization. R code of these functions can be found in the Additional file 2. This section describes these functions and give examples on how to use them.

1.1 Strip-level data retrieval

Function getBeadStrips() retrieves separate strip data from a "LumiBatch" object. This function extracts strip data from a "LumiBatch" object and returns a list with two components which contain data for two strips respectively. Each component is a "LumiBatch" object. An example of using this function is:

```
stripdata <- getBeadStrips(Your_LumiBatch_object)
```

1.2 Strip-level plotting

Function boxplotBeadStrips() shows the boxplot for strip-level data. Data provided to this function should be a "LumiBatch" object [1]. By default, strip-level data are plotted in log2 scale. The first strip in red color and the second strip in green color. Other parameters supplied by users will be passed onto the function boxplot(). An example of using this function is:

```
boxplotBeadStrips(Your_LumiBatch_object, log2=TRUE, names=Your_Strip_Names)
```

1.3 Strip-level normalization

Function normBeadStrips() subsets a "LumiBatch" object by strip and normalizes first strips and second strips separately. Data transformation and between-array normalization functions provided in R/Bioconductor package lumi are utilized by this function [1, 2]. Below is sample code for conducting strip-level normalization:

```
x <- lumiR("Your_Sample_Probe_Profile.txt")        # not background corrected
xn <- normBeadStrips(x, transform.method="log2", normalize.method="quantile")
```

2 Analysis scripts used in this study

The Additional file 3 contains R scripts used for performing the analysis in this study.

To run these scripts, you will need to install R software to your computer. R can be downloaded from http://cran.r-project.org/. You will also need to install limma and lumi packages, which can be downloaded from the Bioconductor project (http://www.bioconductor.org).

Functions in the Additional file 2 will need to be imported into your R environment before running these scripts.

3 Probe filtering

This section gives supplemental figures and plots related to probe filtering.

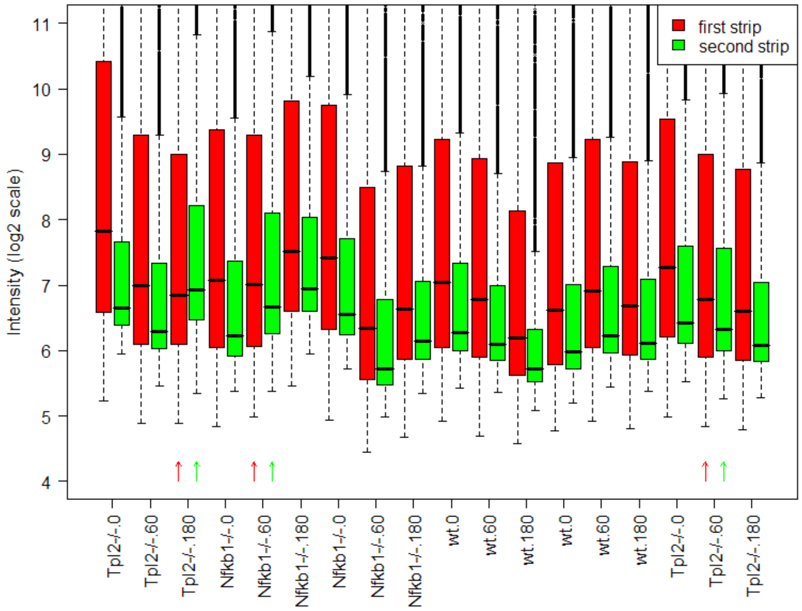

Figure S1: Boxplot showing intensity distributions for each strip on each array for the filtered raw data

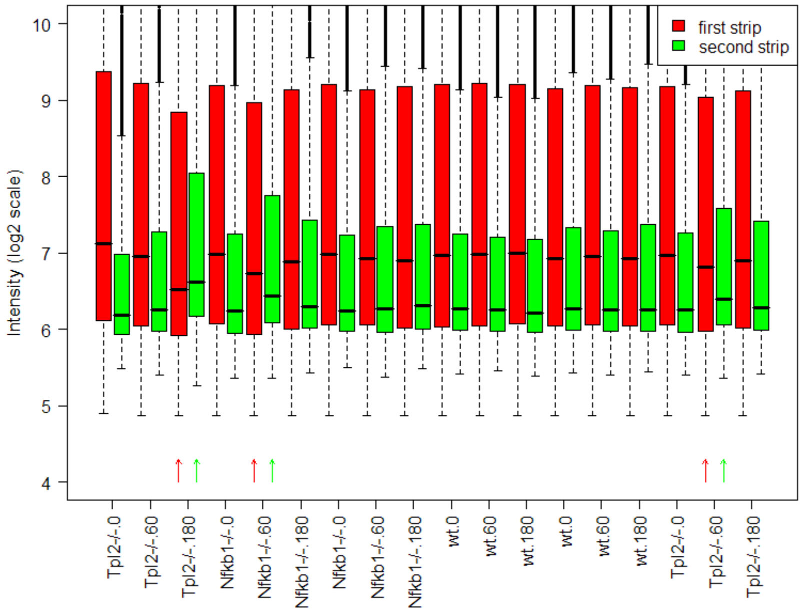

Figure S2: Strip-level intensity distribution boxplot showing the result of array-level normalization on the filtered raw data

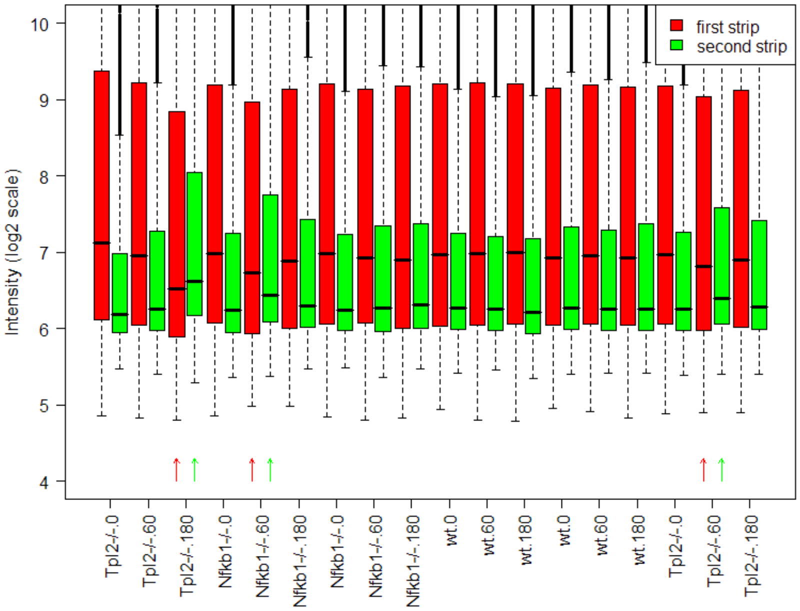

Figure S3: Strip-level intensity distribution boxplot for the filtered array-level normalized data. Array-level normalization was performed on the full raw data. The normalized data were then filtered by removing probes which have detection scores greater than 0.1 on all arrays. This boxplot shows the strip-level intensity distribution for these filtered normalized data.

Table S1: Functional analysis for DE genes at Nfkb1$^{-/-}$ 180 mins obtained from array-level normalization followed by probe filtering

		Array-level		Strip-level	
Source	Term	Count	FDR	Count	FDR
GOTERM_BP_ALL	immune system process	24	3.3E-6	33	8.5E-7
GOTERM_BP_ALL	immune response	19	3.5E-6	25	2.0E-6
SP_PIR_KEYWORDS	cytokine	9	0.012	14	9.0E-5
GOTERM_BP_ALL	cytokine metabolic process	7	0.011	10	4.5E-4
GOTERM_BP_ALL	cytokine biosynthetic process	7	0.013	10	4.8E-4
INTERPRO	Fos transforming protein	3	1.0	5	8.5E-3
GOTERM_BP_ALL	cytokine production	8	0.012	11	1.2E-3
GOTERM_BP_ALL	leukocyte activation	12	0.001	16	2.5E-4
GOTERM_BP_ALL	hemopoietic or lymphoid organ development	10	0.04	14	0.01
GOTERM_BP_ALL	regulation of cytokine biosynthetic process	6	0.039	9	1.1E-3
GOTERM_BP_ALL	immune system development	10	0.05	15	4.4E-3
GOTERM_BP_ALL	cell activation	12	1.4E-3	16	4.1E-4
GOTERM_BP_ALL	hemopoiesis	10	0.021	13	0.016
GOTERM_BP_ALL	positive regulation of translation	6	0.017	7	0.016
GOTERM_BP_ALL	positive regulation of cellular biosynthetic process	6	0.022	7	0.022
KEGG_PATHWAY	Cytokine-cytokine receptor interaction	11	0.049	15	0.011
GOTERM_MF_ALL	cytokine activity	9	0.35	14	0.015
SP_PIR_KEYWORDS	glycoprotein	41	7.4E-3	58	0.011
GOTERM_BP_ALL	lymphocyte activation	10	0.015	14	1.2E-3
GOTERM_BP_ALL	positive regulation of biosynthetic process	6	0.042	7	0.055
GOTERM_BP_ALL	activation of protein kinase activity	6	0.013	6	0.053
GOTERM_BP_ALL	T cell activation	7	0.1	11	3.3E-3
GOTERM_BP_ALL	positive regulation of protein metabolic process	6	0.13	8	0.045

References

1. Du P, Kibbe WA, Lin SM: **lumi: a pipeline for processing Illumina microarray**. *Bioinformatics* 2008, **24**:1547–1548.

2. Lin SM, Du P, Huber W, Kibbe WA: **Model-based variance-stabilizing transformation for Illumina microarray data**. *Nucleic Acids Res* 2008, **36**:e11.
